# Advances in the treatment of recurrent aphthous stomatitis: from synthetic and natural drugs to novel drug delivery systems

**DOI:** 10.3389/fphar.2026.1715554

**Published:** 2026-02-13

**Authors:** Xiangran Kong, Lin Fan, Dawei He, Lin Wang, Jiang Sun

**Affiliations:** 1 Graduate School of Dalian Medical University, Dalian, China; 2 Department of Periodontics and Oral Mucosa Disease, Stomatological Hospital of Dalian University (Dalian Stomatological Hospital), Dalian, China

**Keywords:** drug delivery system, Microneedle, natural drugs, recurrent aphthous stomatitis, synthetic drugs

## Abstract

**Background:**

Recurrent aphthous stomatitis (RAS), the most common inflammatory disease of the oral mucosa, has a complex etiology and diverse pathogenesis. To date, no curative treatment exists. Recurrent oral ulcers severely impair patients' quality of life, making the development of highly effective and safe therapeutic strategies an urgent priority in current clinical research.

**Method:**

Literature references were sourced from publications retrieved via searches on GeenMedical, X-mol, CNKI, and PubMed using the search terms “recurrent aphthous stomatitis” OR “recurrent aphthous ulcer” OR “oral ulcer” AND “treatment” in English and Chinese. The focus was on two dimensions: drug types and delivery systems.

**Results:**

Synthetic drugs such as corticosteroids have clear efficacy, but their potential adverse reactions limit their long-term use. In contrast, natural medicinal components such as licorice and quercetin are gaining increasing attention because of their multitarget mechanisms of action, favorable safety profiles, and therapeutic effects. With respect to delivery systems, novel adhesion agents, microneedles, and nanomedicines currently in development have been evaluated. These advantages include prolonged retention time on ulcer surfaces, enhanced biosafety, improved therapeutic efficacy, and current limitations.

**Conclusion:**

Natural medicines have potential as novel therapeutic options with high efficacy and safety in clinical settings. By enhancing local drug retention and amplifying therapeutic effects, novel delivery systems open new avenues for RAS therapy. Integrating the therapeutic advantages of natural medicines with the precision and sustained-release characteristics of advanced delivery systems represents a key direction for future research.

## 1 Introduction

Recurrent aphthous stomatitis (RAS) is a common oral mucosal disease characterized by recurrent circular or oval oral ulcers that can occur anywhere in the oral cavity, most frequently on non-keratinized epithelium. Epidemiological studies indicate a prevalence ranging from 5% to 66% across different countries (Cheng et al., 2020). RAS is classified into three types based on severity and morphology: minor, major, and herpetiform ulcers. Minor aphthous ulcers constitute 80% of RAS cases, measuring 8–10 mm in diameter. They predominantly affect the buccal mucosa, labial mucosa, and floor of the mouth, with a healing time of 10–14 days. Major aphthous ulcers constitute 10%–15% of RAS cases, with diameters exceeding 1 cm. They commonly occur on keratinized mucosa (lips, soft palate, pharyngeal pillars, gingiva, and dorsal tongue) and typically heal within 6–8 weeks. Herpetiform ulcers present as recurrent clusters of pinpoint (two to three mm) multiple ulcers (up to 100) that heal within 10–14 days (Hasan et al., 2022). The etiology and pathogenesis of RAS are diverse and complex and involve a range of factors, such as genetic factors, food allergens, localized trauma, endocrine alterations, stress and anxiety, certain chemical products, microbial agents, and several systemic diseases (Cheng et al., 2023; Ma and Wang, 2023; Estornut et al., 2024). RAS seriously affects the daily life of patients, severely impairing their nutritional intake, communication, daily activities, and overall quality of life (Ono et al., 2024). Within the diverse therapeutic landscape for RAS, drug therapy has become the most commonly used and dominant clinical treatment option due to its proven efficacy and broad applicability (Shi and Zhou, 2022; Vashishat et al., 2024); In contrast, physical therapy, such as lasers (Amorim dos Santos et al., 2020; Andisheh-Tadbir et al., 2021); microecological regulation, such as probiotics (Cheng et al., 2020; Aggour et al., 2021); and traditional medical therapies, such as acupuncture are more often positioned as valuable adjunctive measures (Yan et al., 2022).

Owing to the complex and diverse etiology and pathogenesis of RAS, there are currently no specific or satisfactory drug therapies available. At present, clinical drug treatment centers on symptom control as the core strategy: (1) alleviating pain; (2) accelerating ulcer healing; and (3) prolonging recurrence intervals. For drug therapy, the topical application of corticosteroids, anesthetics, and antibiotics is recommended as the first-line symptomatic treatment regimen. Systemic administration is considered only in severe cases or when local treatment fails to yield an adequate response (Bhargava et al., 2023). However, existing medications still exhibit significant interindividual variability in efficacy and adverse reactions in some patients, failing to meet the clinical needs of all individuals. While these drugs hold clinical value, they do not produce ideal outcomes for every patient, and severe side effects may occasionally occur in certain individuals (Vitamia et al., 2024). These clinical dilemmas reveal significant research gaps in current treatment strategies.

Given the limitations of first-line therapies, natural Chinese herbal medicines and their active components represent an important class of investigational treatments demonstrating significant potential for application in the treatment of RAS. Traditional Chinese herbal medicines and their active components have a long history in treating oral diseases and exhibit multifaceted biological activities, including antibacterial, antifungal, anti-inflammatory, and antioxidant effects (Dinu et al., 2024; Prayoga et al., 2024). Owing to the widespread use of Chinese herbal medicines and their antibacterial and anti-inflammatory properties, various herbal formulations have been developed for treating RAS (Wen et al., 2021). The ethanol extracts of sage possess antibacterial activity, while its terpenoid compounds exhibit anti-inflammatory effects. Salvizan gel is a pharmaceutical product made from sage extract used to alleviate RAS inflammation. A double-blind clinical trial comparing Salvizan gel and triamcinolone for treating recurrent mild stomatitis demonstrated the significant efficacy of Salvizan gel in RAS treatment, showing marked superiority over triamcinolone in pain recovery and wound healing (Abbasi et al., 2023). A systematic review on the efficacy and safety of topical herbal treatments for RAS indicated that natural herbal applications benefit RAS patients by reducing the ulcer area, shortening the ulcer duration, and alleviating pain without causing severe adverse reactions (Shavakhi et al., 2022).

RAS treatment primarily involves two routes: systemic administration and local mucosal administration (Okur et al., 2021; Yan et al., 2024). Systemic administration faces limitations due to first-pass hepatic metabolism and gastrointestinal enzyme degradation, restricting its widespread application (Sudhakar et al., 2006; Wu et al., 2024). In contrast, local mucosal administration bypasses the digestive tract, avoiding systemic metabolism before reaching the target site, thus demonstrating significant therapeutic advantages. It is currently the preferred method for treating RAS in clinical practice. Commonly used RAS formulations include mouthwashes, powders, ointments, and gels. However, owing to the dynamic moist environment of the oral cavity, existing formulations face limitations in terms of local retention and targeting. Consequently, current research on RAS drug delivery systems is advancing toward novel formulations such as adhesive formulations, microneedles, and nanodevices. These innovations aim to enhance mucosal adhesion and targeted delivery capabilities, thereby further improving therapeutic efficacy.

In summary, this narrative review outlines the types of drug therapies for RAS and advances in local delivery, discusses the limitations of synthetic drugs, and examines the current status and challenges in the research and development of natural active ingredients and novel formulations. This study aims to provide a theoretical basis for future experimental design and clinical practice in RAS management.

## 2 Methods

This review is a narrative literature review. References were sourced from the GeenMedical, X-mol, CNKI, and PubMed databases. The search terms included “recurrent aphthous ulcer” or “recurrent aphthous stomatitis” or “oral ulcer” AND “treatment” in both Chinese and English. The search was conducted up to October 2025. The core literature for this review spans the period from 2019 to 2025. Reference lists from relevant articles were also searched. The literature on drug treatments for RAS has subsequently undergone manual screening and review. The selection criteria included peer-reviewed original research articles, clinical trials, meta-analyses, case reports, and reviews. Letters, abstracts, conference reports, and research protocols were excluded. The review focused on two dimensions: drug types and delivery systems.

## 3 Types of drugs

Drugs for the treatment of RAS can be classified into two main categories: synthetic drugs and natural drugs. Among these, synthetic drugs are widely used as routine clinical treatments, whereas natural drugs have been increasingly emphasized by researchers and clinicians in recent years because of their high safety, significant efficacy, and affordability. A comparison of the therapeutic efficacy of RAS drugs are shown in Table 1.

**TABLE 1:** A comparison of the therapeutic efficacy of RAS drugs.

Drug	Comparator	Dosage	Study design	Overall effect (compared with comparator)			Ref
				Relieve pain	Promote ulcer healing	Recurrence rate	
Lidocaine	Placebo	Topical treatment	Double blind RCT	Positive	N/A	N/A	Descroix et al. (2011)
Doxycycline	Placebo	Topical treatment	Single blind RCT	Positive	Positive	N/A	Vijayabala et al. (2013)
Thalidomide	Prednisone	Systemic treatment	Double blind RCT	Not Significant	Not Significant	Positive	Zeng et al. (2020)
Amlexanox	Triamcinolone acetonide	Topical treatment	Single blind RCT	Negative	Negative	N/A	Kavita et al. (2020)
Licorice	Placebo	Topical treatment	Double blind RCT	Positive	N/A	N/A	Liu et al. (2022a)
Astragalus membranaceus	Placebo	Systemic treatment	RCT	N/A	Positive	Positive	Shao et al. (2015)
Curcumin	Triamcinolone acetonide	Topical treatment	RCT	Negative	Negative	Not Significant	Raman et al. (2020)
Sage (Salvizan gel)	Triamcinolone acetonide	Topical treatment	Double blind RCT	Positive	Positive	N/A	Abbasi et al. (2023)
Rhus coriaria	Triamcinolone acetonide	Topical treatment	Two-arm, single-blind, RCT	Not Significant	Not Significant	N/A	Lavaee et al. (2022)
Cannabidiol	Triamcinolone acetonide	Topical treatment	Double blind RCT	Not Significant	Negative	N/A	Umpreecha et al. (2023)
Aloe vera	Amlexanox	Topical treatment	Double blind RCT	Positive	Positive	N/A	Yousef et al. (2022)
	Triamcinolone acetonide	Topical treatment	RCT	Not Significant	Not Significant	N/A	Tariq et al. (2024)
Kaempferia galanga L	Triamcinolone acetonide	Topical treatment	Vivo study (Wistar rats)	N/A	Positive	N/A	Wahyuni et al. (2022)
Quercetin	Benzydamine hydrochloride	Topical treatment	Double blind RCT	Not Significant	Positive	N/A	Pandya et al. (2017)

RCT, randomized controlled trial.

### 3.1 Synthetic drugs

Synthetic drugs are drugs with specific pharmacological activities that are prepared using chemical synthesis (Vitamia et al., 2024). In the clinical treatment of RAS, the commonly used synthetic drugs can be classified into the following categories: corticosteroids, anesthetics, antimicrobial agents, and immunomodulators. Table 2 provide the dosages, mechanisms, advantages, and adverse events of various synthetic drugs used for treating RAS.

**TABLE 2:** Dosages, mechanisms, advantages, and adverse events of various synthetic drugs for treating RAS.

Types of synthetic drugs	Commonly used drugs	Dosage	Mechanism	Advantages of efficacy	Adverse events	Ref
Corticosteroids	Triamcinolone acetonide	Topical (0.05%–0.5%3–10 v/d)	Anti-inflammatory mechanism:inhibits the NF-κB signaling pathway, thereby effectively suppressing COX-2 activity and tumor necrosis factor-α(TNF-α) synthesis	Characterized by rapid action and high efficiencys	Long-term use may alter the oral microbiota, potentially leading to mucosal atrophy, allergic reactions, and oral candidiasis	Ono et al. (2024), Lau and Smith. (2022)
	Prednisone	Systemic (25 mg/day)				
Anesthesia	Lidocaine	Topical (2% spray or gel)	Pain relief mechanism:reversibly block voltage-gated sodium channels	Significantly enhance the analgesic efficacy of ulcer lesions	Causes a reduction in pain sensitivity in healthy mucosal areas, leading to loss of taste sensation and mucosal damage during chewing. No significant therapeutic effect on promoting ulcer healing	Prathoshini et al. (2020)
Antibiotics	Minocycline	Topical (0.5% mouthwash)	Binds to the 30S ribosomal subunit of bacteria, inhibiting bacterial protein synthesis to achieve bacteriostatic effects. Tetracycline antibiotics can inhibit the activity of collagenase and gelatinase	Shortened ulcer healing time and control of daily ulcer pain	Long-term use of antimicrobial agents may lead to bacterial resistance, which could further exacerbate the adverse effects of microorganisms	Skučas et al. (2023)
	Doxycycline	Topical (low doses)				
	Penicillin G potassium	Systemic (50 mg pills 4 v/d 4 days)				
Immunomodulators	Thalidomide	Systemic (25 mg/d)	Inhibit the production of TNF-α	Effectively reduce the frequency of ulcer recurrence	Embryo-fetal toxicity, teratogenicity, thromboembolic disorders, and peripheral neuropathy	Jian et al. (2024)
	Levamisole	Systemic (150 mg three times a week during 6 months)				
Others	Amlexanox	Topical (5%ointment 2–4 v/d)	Unclear	It is the only FDA-approved prescription medication for the treatment of RAS	Unclear	Belenguer-Guallar et al. (2014)

(v/d = times a day).

#### 3.1.1 Corticosteroids

**FIGURE 1:**
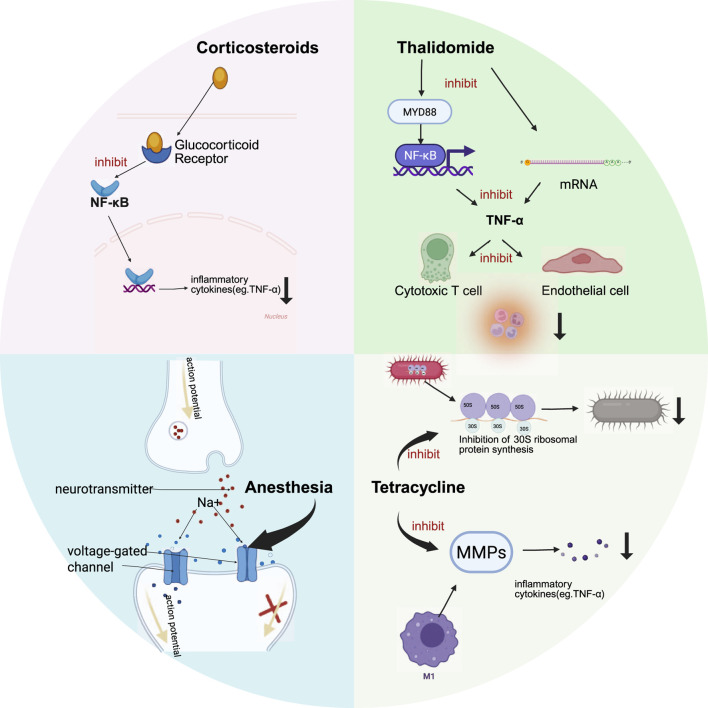
Mechanism Diagram of Synthetic Drugs (Corticosteroids, Anesthesia, Antibiotics (Tetracycline), Immunomodulators (Thalidomide) Treating RAS Glucocorticoids activate cellular glucocorticoid receptors, suppressing the NF-κB signaling pathway to inhibit TNF-α production while promoting anti-inflammatory factors. Anesthetics inhibit the generation and conduction of action potentials in nerve endings by reversibly blocking voltage-gated sodium channels. Tetracycline exerts antimicrobial effects through bacterial protein synthesis inhibition and suppresses matrix metalloproteinases that promote ulcer progression. Thalidomide inhibits TNF-α by suppressing the NF-κB signaling pathway and mRNA expression levels. Created in BioRender. Xiangran, K. (2026) https://BioRender.com/ju92wlm.

Corticosteroids are widely used as first-line treatments because of their potent anti-inflammatory effects and are commonly employed to manage oral mucosal diseases such as RAS lichen planus (Bhargava et al., 2023; Hasan et al., 2019). The first-line clinical application of steroids used currently for RAS involves the activation of the intracellular glucocorticoid receptor, which prompts the receptor in the cell nucleus to upregulate the expression of a variety of target genes to inhibit the nuclear factor kappa-B (NF-κB) signaling pathway, thereby effectively inhibiting cyclo-oxygenase-2 (COX-2) activity and the biosynthesis of key proinflammatory mediators such as tumor necrosis factor-α (TNF-α), ultimately achieving anti-inflammatory therapeutic objectives. By effectively blocking the inflammatory cascade, they suppress pain caused by ulcerative stomatitis and inhibit mechanical sensitivity in peripheral nerves, significantly alleviating ulcer-related pain (Naniwa et al., 2021). The corticosteroids used to treat RAS include triamcinolone acetonide, dexamethasone, fluocinolone acetonide, and clobetasol, which significantly reduce the size of the ulcer and promote the mucosal repair process (Lau and Smith, 2022). Triamcinolone acetonide is widely used in clinical applications owing to its significant anti-inflammatory and ulcer-healing effects and is often reported as a representative drug in comparative studies of therapeutic efficacy with various types of drugs. The use of corticosteroids is more effective in the early stages of RAS flare-ups. However, topical treatment alone does not reduce the formation of new ulcers. For patients with severe RAS or those with frequent mild RAS flare-ups, it may be insufficient to achieve the ultimate goal of improving the quality of life for RAS patients. Systemic corticosteroid therapy effectively reduces recurrence rates; however, the severe adverse effects associated with long-term systemic corticosteroid use may outweigh the benefits for RAS patients (Shi and Zhou, 2022) (Figure 1).

#### 3.1.2 Anesthetics

Local anesthetic drugs are the core therapeutic agents used for pain management in patients with RAS. These drugs provide rapid pain relief by reversibly blocking voltage-gated sodium channels and inhibiting the generation and conduction of action potentials in nerve endings. The anesthetics used to treat RAS include lidocaine and benzocaine. Among these, the amide-type local anesthetic lidocaine has become the first-line clinical choice because of its rapid onset of action and significant analgesic effect (Bhargava et al., 2023). Altenberg et al. demonstrated that the topical application of 2% lidocaine solution significantly enhanced analgesic efficiency in patients with ulcer lesions (Altenburg et al., 2007). Notably, the role of local anesthetics (such as lidocaine) in RAS treatment is currently limited to symptomatic pain relief; there is insufficient evidence to suggest that they can alter disease progression, promote ulcer healing, or prevent recurrence (Figure 1).

#### 3.1.3 Antibiotics

The use of antibiotics is reportedly helpful in the management of RAS (Lau and Smith, 2022). Topical application of antibiotics, including tetracyclines (e.g., doxycycline, minocycline) and penicillin preparations, can result in lower pain scores, smaller ulcers, and a significant reduction in the healing cycle (Skučas et al., 2023). Antibiotics exhibit broad-spectrum bacteriostatic effects. Tetracyclines inhibit bacterial protein synthesis by binding to the 30S ribosomal subunit of both Gram-positive and Gram-negative bacteria. This achieves bacteriostatic effects. The mechanism for treating RAS extends beyond antibacterial action. Matrix metalloproteinases (MMPs) are primary enzymes responsible for degrading the extracellular matrix. Tetracycline drugs have been demonstrated to inhibit the activity of collagenase and gelatinase, particularly MMP-9, thereby potentially suppressing inflammatory cytokines to achieve anti-inflammatory effects (Piacentini et al., 2019) Figure 1.

#### 3.1.4 Immunomodulators

Immunomodulators are effective systemic agents used for the treatment of refractory oral ulcers. The immunomodulators used to treat RAS include levamisole and thalidomide (Prajapat et al., 2021). Tumor necrosis TNF-α levels in both peripheral blood and saliva of RAS patients are significantly higher than in healthy individuals. This event stimulates cytotoxic T lymphocytes and increases endothelial cell expression, leading to the migration of inflammatory cells to the site of inflammation and resulting in ulcer development. Thalidomide can block TNF-α expression through various possible mechanisms. One such mechanism involves inhibiting the expression of NF-κB, which functions as a transcription factor for TNF-α. Another study by Noman, A.S. et al. demonstrated that thalidomide suppresses lipopolysaccharide-induced TNF-α production by downregulating the MyD88 protein and mRNA (Noman et al., 2009; Hussain et al., 2022). A randomized controlled clinical trial compared the efficacy of systemic thalidomide with that of corticosteroids. Although both groups exhibited similar levels of pain, ulcer counts, and healing times, patients receiving thalidomide demonstrated longer intervals between RAS recurrence. Notably, while immunomodulators are used to treat RAS, their use is restricted by potential side effects, including embryo-fetal toxicity, teratogenicity, thromboembolic disorders, and peripheral neuropathy. Thalidomide is contraindicated in pregnant or potentially pregnant women (Prajapat et al., 2021; Deng et al., 2022; Jian et al., 2024). A randomized controlled trial demonstrated that 25 mg/day thalidomide provides favorable long-term efficacy in prolonging the recurrence interval of RAS, with acceptable safety (Figure 1).

#### 3.1.5 Other synthetic drugs

Amlexanox is one of the most widely studied topical drugs for the treatment of RAS and has anti-inflammatory and antiallergic effects (Belenguer-Guallar et al., 2014). Vitamin and micronutrient supplements are also essential for the prevention and treatment of RAS, allowing for effective control of pain and promoting ulcer healing (Cui et al., 2022). Recombinant human epidermal growth factor, an endogenous growth factor that promotes wound repair and healing, has been widely used in wound healing (Yu et al., 2024). The potential effects of naltrexone on scar formation and immunomodulation favor oral ulcer healing (Diaz et al., 2024).

Rebamipide is a novel mucosal protective agent that maintains viable epithelial cells and repairs damaged tissue through a multimodal mechanism (Akagi et al., 2019). The published literature has demonstrated the efficacy of rebamipide in the pharmacological treatment of Behçet’s disease and RAS (Kudur and Hulmani, 2013). A randomized controlled clinical trial by Hasan et al. demonstrated that 5% Amlexanox paste and rebamipide tablets significantly outperformed analgesic and antiseptic gel-Dologel CT in reducing the ulcer area, erythema, and pain (Hasan et al., 2022).

### 3.2 Natural drugs

**FIGURE 2:**
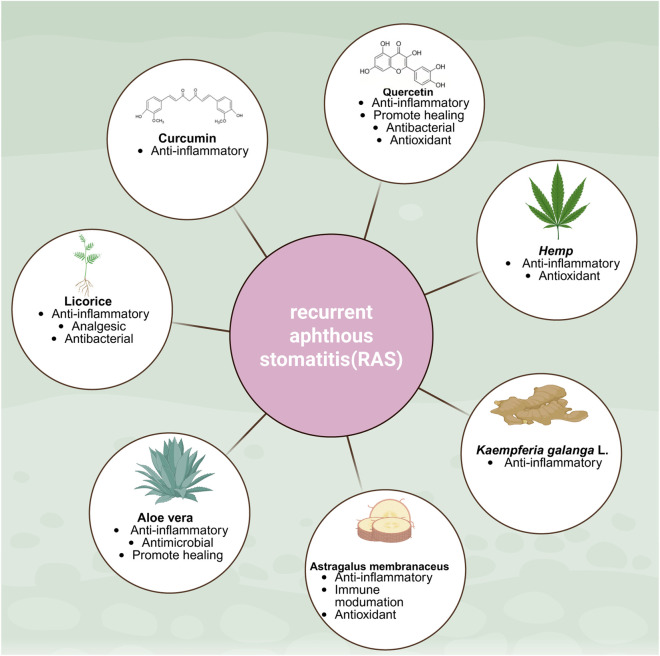
Herbal medicines and pharmacological effects in the treatment of recurrent aphthous stomatitis. Created in BioRender. Xiangran, k. (2026) https://BioRender.com/9w61o9k.

In recent years, natural medicines have been increasingly gaining widespread attention in the field of RAS treatment, demonstrating unique therapeutic advantages (Ayoub et al., 2021; El-Zahar et al., 2022; Molania et al., 2022; Sanguansajapong et al., 2022; Shavakhi et al., 2022; Vitamia et al., 2024). Currently, certain traditional Chinese patent medicines derived from natural sources, such as Kangfu Xin Liquid and Watermelon Frost, are widely used in clinical practice. These formulations exhibit no toxic side effects, high safety profiles, alleviate RAS symptoms, and promote ulcer healing. Moreover, numerous herbal medicines and their active components continue to demonstrate significant therapeutic potential. Current research hotspots mainly include plant-derived herbal medicines and their active ingredients (Dinu et al., 2024; Prayoga et al., 2024). Research in this field has inherited the long history of traditional Chinese medicine and combines it with modern pharmacological research methods (Zhai et al., 2025). Traditional Chinese medicines with significant potential in treating RAS include licorice, aloe vera, astragalus, galangal, hemp seed, curcumin, and quercetin. These compounds exert synergistic effects through multiple pathways—including anti-inflammatory and antioxidant actions, as well as promoting mucosal repair—offering additional natural options for the prevention and treatment of RAS (Figure 2). Table 3 shows natural Chinese herbal medicines, active ingredients, mechanism of action, dosage form, clinical or preclinical trial phase, advantages and limitations.

**TABLE 3:** Natural Chinese herbal medicines, active ingredients, mechanism of action, dosage form, clinical or preclinical trial phase, advantages and limitations.

Herbal	Compound or active ingredient	Potential mechanisms	Form	Clinical or preclinical trials	Advantages of efficacy	Limitation	Ref
Licorice	Isoliquiritigenin, glycyrrhizic acid	Anti-inflammatory mechanism: Reduction of prostaglandin E2 (PGE2), matrix metalloproteinases (MMPs), tumor necrosis factor (TNF), and free radicals Analgesic mechanism: Blocks action potential conduction by affecting NAV channels in peripheral nociceptive fibers Antimicrobial mechanism: Inhibits gram-positive and gram-negative bacteria	Juice, hydrogel	Preliminary clinical study	Rapidly relieve pain and shorten healing time	Consuming large amounts can temporarily stain teeth and tongue, leading to tooth decay	Liu et al. (2022b), AlDehlawi and Jazzar. (2023)
Aloe vera	Glucomannan, Gibberellin, acemannan	Anti-inflammatory mechanism: Enhances anti-inflammatory effects by suppressing the production of reactive oxygen species metabolites, thereby preventing oxidative stress Promotion of healing mechanisms: Enhances collagen synthesis, stimulates fibroblast activity and proliferation Antimicrobial mechanism: Reduces the abundance of harmful oral bacteria, including *Actinomyces*, *Granulomonas*, and *Peptostreptococcus*	Gel	Preliminary clinical study	Aloe vera gel outperforms traditional medications (such as amlexanox oral paste) in promoting ulcer healing and alleviating pain, while also being better tolerated by patients	Differences in geographical location, plant composition, extraction methods, and sample preparation techniques result in variations in the chemical composition and biological activity of aloe vera	Yousef et al. (2022), Kumar et al. (2019)
Astragalus membranaceus	Astragalus polysaccharide	Immune modulation mechanism: Promotes immune cell proliferation and stimulates cytokine release Anti-inflammatory mechanism: Inhibits endotoxin-stimulated mitogen-activated protein kinase (MAPK) and nuclear factor-κB (NF-κB) inflammatory pathways Antioxidant mechanism: Restores mitochondrial membrane potential in human umbilical vein endothelial cells (HUVECs) and reduces reactive oxygen species (ROS) levels	Injection, Mouthwash	Preliminary clinical study	Systemic administration of astragalus membranaceus injection significantly reduces the recurrence rate of ulcers in RAS.	Further investigation into its biological activity and pharmacological effects is needed	Shao et al. (2015), Zou et al. (2018)
Kaempferia Galanga L	Kaempferol, ethyl p-methoxycinnamate, and ethyl cinnamate	Anti-inflammatory mechanism: Interleukin-6 and prostaglandins exhibit inhibitory activity by suppressing PGH2 formation through the COX signaling pathway	Gel	Vivo study (Wistar rats)	Compared with triamcinolone acetonide, topical EEKG more effectively improves ulcer area recovery rate and inflammatory signs	Lack of clinical and safety trials	Wahyuni et al. (2022)
Hemp	cannabidiol	Anti-inflammatory mechanism: Reduces the release of inflammatory mediators such as TNF-α and IL-6 Antioxidant mechanism: CBD reduces the production of reactive oxygen species (ROS)	Paste	Preliminary clinical study	CBD exhibits anti-inflammatory effects in the early stages of RAS and analgesic effects in the late stages	The detailed regulatory mechanisms of CBD remain unclear, and further animal studies and clinical trials are needed to assess the final outcomes	Umpreecha et al. (2023)
Turmeric	Curcumin	Anti-inflammatory mechanism: Inhibits the activity of inflammatory mediators such as COX-2,5-lipoxygenase (5-LOX), and nitric oxide synthase (NOS), while also significantly downregulating the expression levels of pro-inflammatory cytokines including TNF-α and interleukin-6 (IL-6)	Gel	Preliminary clinical study	Curcumin and triamcinolone showed comparable efficacy in terms of pain, size, healing time, recurrence frequency, and ulcer count, with curcumin gel demonstrating superior tolerability	Poor solubility May cause contact dermatitis, gastrointestinal issues, and yellow pigment easily adheres to the tongue surface	Raman et al. (2020), Dinu et al. (2024)
Huang Bai Agarwood et al.	Quercetin	Anti-inflammatory mechanism: Inhibits excessive expression of inflammatory cytokines and blocks the activity of inflammation-related enzymes Antioxidant mechanism: Directly scavenges free radicals to mitigate oxidative stress damage Antimicrobial mechanism: Exerts antibacterial effects by disrupting bacterial membrane structures Promotes healing mechanism: Quercetin also inhibits fibrosis, reduces scar formation, and promotes fibroblast proliferation	Gel	Preliminary clinical study	Quercetin gel significantly outperforms phenylephrine hydrochloride mouthwash in promoting ulcer healing and effectively reduces ulcer area	Poor solubility Clinical and safety trials are scarce; more research should be conducted	Pandya et al. (2017), Dinu et al. (2024)

#### 3.2.1 Licorice

Licorice is one of the herbs with the longest history of application in traditional Chinese medicine and has demonstrated significant clinical efficacy in treating RAS and traumatic ulcers (AlDehlawi and Jazzar, 2023; Moussa et al., 2023). Licorice has a wide range of pharmacological effects, including anti-inflammatory, anti-infective, antiulcer, and antitumor activities (Yang et al., 2017). Most bioactive compounds identified in licorice are triterpenoids or flavonoids, both of which exhibit antiulcer pharmacological effects. Isoliquiritigenin, a flavonoid extracted from licorice, exerts its anti-ulcer analgesic action by blocking action potential conduction and modulating the voltage-gated sodium channels in peripheral nociceptive fibers (Miyamura et al., 2021). The primary triterpenoid compound, glycyrrhizin acid, has a multitarget pharmacological mechanism for treating oral ulcers, and its efficacy has been extensively validated by modern pharmacological research and clinical practice. Glycyrrhizin acid reduces inflammatory responses by decreasing the levels of prostaglandin E2 (PGE2), matrix metalloproteinases (MMPs), tumor necrosis factor (TNF), and free radicals. The plant active ingredients, represented by the aqueous and ethanolic extracts of *Glycyrrhiza glabra*, have shown unique antimicrobial advantages; not only can they block biofilm formation by inhibiting the bacterial population-sensing system, but they also target the fungal morphology transition pathway to inhibit the yeast-mycelium phase transition while significantly reducing the expression of virulence factors, such as α-hemolysin, thus exerting a dual inhibitory effect on both Gram-positive and Gram-negative bacteria (e.g., *E. coli*) (AlDehlawi and Jazzar, 2023). Nasry et al. conducted a randomized clinical trial involving 60 subjects. They reported that, after treatment with a licorice-containing adhesive, the pain score and ulcer surface area were significantly lower than those of the control group (Nasry et al., 2016). A randomized, double-blind, placebo-controlled trial evaluating licorice juice for the relief of aphthous ulcers used a 10-point visual analog scale (VAS) to assess patients' pain levels before and after treatment. Pain levels decreased in both the licorice group and the placebo group on both Day 1 and Day 2, but the reduction in pain levels was greater in the licorice group than in the placebo group. Licorice juice rapidly alleviates pain without causing any discomfort (Liu H. L. et al., 2022). Licorice provides prompt pain relief in managing RAS and significantly promotes ulcer healing.

#### 3.2.2 Aloe vera

Aloe vera is a medicinal plant that has multiple pharmacological activities and is widely used for the treatment of oral mucosal diseases, such as oral lichen planus and radiation-induced mucositis. The anti-inflammatory, antimicrobial, and antioxidant properties of aloe vera have significant therapeutic effects on RAS (Gao et al., 2019; Zou et al., 2022). Aloe vera enhances anti-inflammatory effects by inhibiting the production of reactive oxygen metabolites, thereby preventing oxidative stress. Beta-glucans (a polysaccharide rich in mannose) and gibberellin (a growth hormone) in aloe extract promote collagen synthesis and the activity and proliferation of fibroblasts. Aloe also contains a range of components, such as acetylmannan, which stimulate factors such as fibroblasts and collagen that aid in wound repair. This promotes the repair process and proliferation of epithelial cells, increasing their potential for wound healing. Aloe vera gel also influences the healing process of recurrent oral ulcers and the normal microbial flora. It can reduce the abundance of harmful oral bacteria, including *Actinomyces*, *Granulomonas*, and *Peptostreptococcus* (Kumar et al., 2019; Shi et al., 2020). Tariq et al. compared the clinical efficacy of aloe vera gel versus 0.1% triamcinolone for treating mild RAS. Intergroup comparisons revealed no significant differences in ulcer area reduction, pain VAS scores, or burning sensations. Both aloe vera and 0.1% triamcinolone were found to be equally effective in reducing ulcers, pain, and burning sensations associated with RAS (Tariq et al., 2024). A three-arm randomized controlled clinical trial comparing the efficacy of aloe vera gel versus 5% amlexanox oral paste for RAS treatment evaluated ulcer area reduction and recorded pain at the initial visit. Compared with the conventional oral paste amlexanox, aloe vera gel demonstrated superior efficacy in promoting ulcer healing and alleviating pain, with better patient tolerability. It is particularly suitable for children, pregnant women, and immunocompromised individuals (Yousef et al., 2022).

#### 3.2.3 Astragalus membranaceus

Astragalus is a tonic drug commonly used in Chinese medicine clinics, with demonstrated efficacy in the treatment of oral mucosal and periodontal diseases (Zou et al., 2018). The pharmacological effects of this drug include immunomodulation, antiviral effects, and the promotion of tissue repair (Fu et al., 2014; Ren et al., 2023). The primary components of Astragalus are polysaccharides, flavonoids, and saponins. *Astragalus polysaccharides* are water-soluble heteropolysaccharides that promote ulcer healing through multiple mechanisms. They regulate immune function by enhancing immune cell proliferation, stimulating cytokine release, influencing immunoglobulin (Ig) secretion, and modulating immune signaling. Astragalus polysaccharide extract (APS) mitigates hydrogen peroxide-induced damage by restoring the mitochondrial membrane potential in human umbilical vein endothelial cells (HUVECs) and reducing reactive oxygen species (ROS) levels. Additionally, *Astragalus polysaccharides* suppress the production of inflammatory and chemotactic factors by inhibiting the activation of the mitogen-activated protein kinase (MAPK) and nuclear factor-κB (NF-κB) inflammatory pathways induced by endotoxin stimulation (Zhang Q. et al., 2025). Hastana selected 12 clinical cases of chronic kidney disease with recurrent oral ulcers where other treatments had failed to produce significant efficacy. Patients were instructed to rinse with Astragalus solution 3–4 times daily while applying it to ulcer surfaces with cotton swabs. Following treatment, ulcer areas decrease, pain subsides, healing accelerates, and recurrence rates decrease (Hastana, 2012). A randomized controlled clinical trial evaluating Astragalus injection for treating RAS demonstrated that both the observation group and the Astragalus injection group presented significantly lower scores for local symptoms and signs at 1 week and 1 month post-treatment than at baseline (P = 0.000). The 1-year cumulative recurrence rate was lower in the Astragalus injection group than in the control group (P = 0.004), indicating that Astragalus injection significantly promotes ulcer healing and delays recurrence in RAS patients (Shao et al., 2015).

#### 3.2.4 Kaempferia galanga L

*Kaempferia galanga* L. (KG) has potential value in RAS therapy as an emerging botanical medicine because of its anti-inflammatory, analgesic, and antioxidant effects (Pathan et al., 2024). Phytochemical screening of galangal revealed that kaempferol, ethyl p-methoxycinnamate, and ethyl cinnamate exhibit superior anti-inflammatory and anti-injury activities compared with those of indomethacin. KG has been shown to have inhibitory effects on several inflammatory marker proteins, including interleukin-6 and prostaglandins. Furthermore, KG suppresses PGH2 formation via the COX signaling pathway. A study on the anti-inflammatory effects and wound healing effects of different concentrations of KG ethanol extract (EEKG) in chemically induced oral mucosal ulcers in Wistar rats was performed, and the ulcer area recovery rate, inflammatory sign recovery rate, and histopathological score were used as indicators. The results revealed that 0.5% EEKG effectively increased the ulcer area recovery rate and reduced inflammatory sign scores. Doses ranging from 0.5% to 2% EEKG significantly reduced histopathological scores. Compared with triamcinolone, locally applied EEKG demonstrated superior efficacy (Wahyuni et al., 2022). These findings provide important evidence for the clinical translation and application of KG.

#### 3.2.5 Hemp

Hemp is one of the oldest cultivated plants and has unique medicinal properties. Cannabidiol (CBD) is currently the most extensively studied multitarget nonpsychoactive cannabinoid and is a non-intoxicating component of hemp (Kongkadee et al., 2022). CBD functions as an effective antioxidant and anti-inflammatory agent in oral ulcers. A study on the protective effect of cannabidiol against chemotherapy-induced oral mucositis in mice via the Nrf2/Keap1/ARE signaling pathway revealed that CBD reduces local inflammatory responses by suppressing the production of high levels of inflammatory cytokines tumor necrosis factor-α and IL-6. It mitigates oxidative stress-induced damage to the oral mucosa by reducing reactive oxygen species (ROS) production. Experiments have demonstrated that CBD treatment significantly reduces ROS levels in oral epithelial cells while enhancing antioxidant enzyme activity (Li et al., 2022). A randomized controlled clinical trial evaluating the topical application of 0.1% cannabidiol (CBD) assessed the clinical safety and efficacy of 0.1% CBD treatment for RAS through measurements of ulcer and erythema size, pain scores, and satisfaction ratings. Results indicate that topical application of 0.1% CBD safely and effectively promotes RAS ulcer healing, as evidenced by reduced ulcer area. Given its favorable safety profile and dual therapeutic effects, this formulation offers a viable treatment option for RAS patients who refuse topical steroids (Umpreecha et al., 2023).

#### 3.2.6 Curcumin

Curcumin is a polyphenolic compound and the main phytochemical of turmeric (Shi et al., 2023), which reportedly has potent analgesic, anti-inflammatory, antioxidant, and antimicrobial properties (Liu S. et al., 2022). Curcumin is used to treat a variety of systemic diseases and oral mucosal diseases (Rai et al., 2021; Rai et al., 2022). Curcumin not only inhibits the activity of inflammatory mediators such as COX-2,5-lipoxygenase (5-LOX), and nitric oxide synthase (NOS) but also significantly downregulates the expression levels of pro-inflammatory cytokines such as TNF-α and interleukin-6 (IL-6). This multidimensional regulation of the inflammatory cascade confers unique therapeutic advantages in promoting mucosal repair following radiotherapy-induced oral mucositis (Ramezani et al., 2023). Curcumin reportedly effectively relieves pain and shortens the healing time of RAS patients, thereby significantly shortening the healing time of the lesions and significantly reducing lesion size. Curcumin can shorten the ulcer healing time through its antioxidant and antimicrobial properties (Al-Maweri et al., 2022; Ramezani et al., 2023). Clinical studies have shown that the efficacy of curcumin topical gel is comparable to that of topical triamcinolone acetonide in the treatment of RAS, whereas curcumin gel is better tolerated, especially for children, pregnant women, lactating women, immunocompromised individuals, and other special populations, demonstrating good clinical application value (Raman et al., 2020).

#### 3.2.7 Quercetin

**FIGURE 3:**
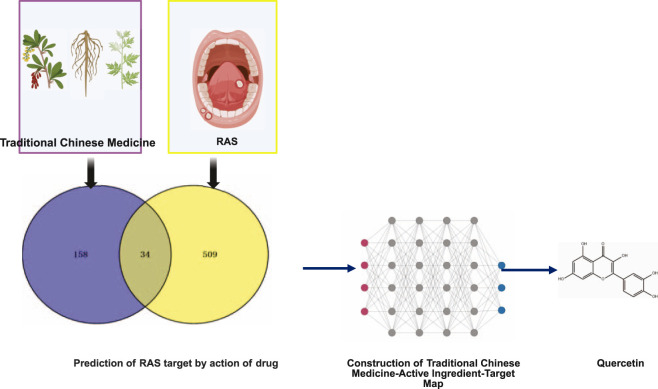
Framework of quercetin for network pharmacological screening of herbal medicines with RAS. Created in BioRender. Xiangran, k. (2026) https://BioRender.com/r7xx61f.

Quercetin is a potent natural flavonoid compound that is widely found in a variety of Chinese herbal medicines (Batiha et al., 2020), such as Astragalus (Zhong et al., 2023), Huang Bai (Tang et al., 2022), and agarwood (Tao et al., 2024), as well as in compound preparations such as licorice laxative heart soup (Yuan et al., 2024). Studies have shown that quercetin has a variety of pharmacological activities, such as antioxidant, anti-inflammatory, antibacterial, and antiviral activities, and plays a significant role in the treatment of RAS, allergies, metabolic diseases, and cardiovascular diseases (Alizadeh and Ebrahimzadeh, 2022). In recent years, network pharmacology has been used as an important research tool for elucidating the multicomponent-multitarget synergistic mechanism of action of herbal medicines (Yang et al., 2025). Through network pharmacology analysis, quercetin was found to be the core active ingredient in several herbal compounds for the treatment of RAS, such as the licorice laxative Tang. Network pharmacological studies have revealed close associations of quercetin with key inflammatory factors, such as IL-6, TNF, and IL-1β (Table 4) (Wang L. L. et al., 2024). Quercetin can reduce the overexpression of inflammatory cytokines and inhibit inflammation-producing enzymes, protect the body from reactive oxygen species by directly scavenging free radicals, and disrupt bacterial membranes, leading to the destruction of bacterial surfaces and internal structures, exerting antimicrobial activity, promoting wound healing by decreasing fibrosis, restricting scarring, and promoting fibroblast proliferation (Chaudhary et al., 2025). A comparison of the effects of quercetin gel and benadryl hydrochloride gargle on ulcer healing revealed that, compared with benzydamine hydrochloride, quercetin significantly reduced ulcers, and the topical application of quercetin gel resulted in beneficial results in patients with mild RAS (Pandya et al., 2017). Quercetin-containing soluble microneedle patches that are biocompatible with human skin keratinocytes and exhibit potent anti-inflammatory effects for wound healing have been prepared (Anjani et al., 2025). Quercetin is a safe, well-tolerated, and effective treatment that promotes the complete healing of ulcers within a short period (Figure 3) (Doersch and Newell-Rogers, 2017; Beken et al., 2020; Mi et al., 2022).

**TABLE 4:** Pharmacological analysis of quercetin treatment for the RAS network.

Components	Single drug	Function	Core targets	Ref
Quercetin	Astragalus membranaceus	Anti-inflammatory effect Antioxidant effect	IL6, IL1B, TNF, CCL2	Zhong et al. (2023)
Quercetin	Huang Bai	Anti-inflammatory effect Antioxidant effect	TNF, MMP3	Tang et al. (2022)
Quercetin	Agarwood	Anti-inflammatory effect	IL6, TNF, IL1β, CCL2	Tao et al. (2024)
Quercetin	Gan Lu Yin	Anti-inflammatory effect Antioxidant effect	IL- 6, TNF, AKT1, TP53, VEGFA, IL-1β	Wang et al. (2024a)
Quercetin	Gancao Xiexin decoction	Anti-inflammatory effect	IL-6, TNF, IL-1β, ALB	Yuan et al. (2024)
Quercetin	Zhibai Dihuang Pill	Anti-inflammatory effect Antioxidant effect	IL-6, TNF, IL-1β, ALB, CCL2, IL-10, VEGFA, MMP9, VCAM1, ICAM1	Peng (2023)

## 4 Topical drug delivery system

**FIGURE 4:**
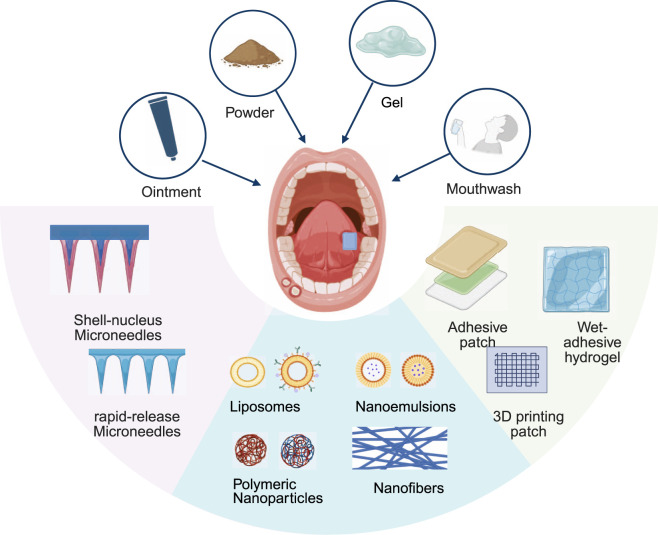
Schematic of topical drug delivery. Created in BioRender. Xiangran, k. (2026) https://BioRender.com/itnbyr4.

Topical mucosal administration is currently the preferred clinical approach for treating RAS (Dubashynskaya et al., 2024). Compared with systemic administration, oral mucosal delivery offers distinct advantages: the thin keratinized epithelium of the oral mucosa, with its dense vascular network and ease of access, provides an ideal interface for drug delivery. Furthermore, local administration effectively circumvents the reduced efficacy caused by first-pass metabolism and minimizes systemic side effects resulting from excessive drug use (Liu et al., 2025). Currently, commonly used RAS formulations include mouthwashes, powders, ointments, and gels. However, owing to the dynamic moist environment of the oral cavity, existing formulations face limitations in terms of local retention and targeting. To overcome these shortcomings, researchers have increasingly developed more efficient novel delivery systems using animal models in recent years. Consequently, current RAS drug delivery system research is advancing toward novel formulations such as adhesive preparations, microneedles, and nanomedicines. These approaches aim to enhance therapeutic efficacy by improving mucosal adhesion and targeted delivery capabilities (Figure 4). Table 5 summarizes local drug delivery systems, dosage forms, advantages, research and development phases, and limitations.

**TABLE 5:** Local drug delivery systems, dosage forms, advantages, research and development phase, limitations.

Drug delivery system	Dosage forms	Advantages of drug delivery system	Limitation	Research and development phase	Ref
Mouthwash	Liquid	Its fluid characteristics enable it to cover all areas of the oral cavity, making it easy to use	Not readily retained at the oral surface Uncontrolled and inconsistent drug delivery Long-term use may cause adverse reactions such as taste abnormalities and temporary staining of teeth and tongue surface, and may disrupt the normal balance of oral flora	Clinical application	Ni et al. (2022)
Powders	Solid	Targeted treatment, high local drug concentration, and rapid onset of action	The ulcer site is easily washed away by saliva, leading to a decrease in drug concentration	Clinical application	Wu et al. (2024)
Ointment	Semi-solid	Apply directly to the ulcer surface to form a physical barrier	Poor adhesion, short drug retention time, requiring frequent administration	Clinical application	Suharyani et al. (2021)
Gel					
Adhesive agent	Semi-solid、 Solid	Increase the duration of drug contact with the ulcer to achieve sustained-release of the medication at the local site	A small portion of clinically used adhesion agents are diluted or washed away by saliva within 2–3 h, while the majority remain in the laboratory stage	Mostly animal studies Limited clinical applications	Zhang et al. (2024a), Zhang et al. (2025a)
Microneedle	Solid	Creates micron-level pathways, significantly enhancing transdermal drug absorption rates, and can simultaneously carry multiple drugs	Current research is limited to animal studies	Animal study	Kong et al. (2024), Liu et al. (2021)
Nanomaterials	​	Nanoformulations significantly enhance therapeutic efficacy due to their superior drug-loading capacity, *in vivo* stability, and controlled release properties	Research on oral mucosal delivery systems based on nanomedicine technology remains in the fundamental research stage, lacking systematic comprehensive quality evaluation	Animal study	Chen et al. (2023b)

### 4.1 Mouthwash

Mouthwash is a commonly used formulation in RAS treatment. Its fluid characteristics enable it to cover all areas of the oral cavity, effectively reducing the oral bacterial load and alleviating inflammation (Ni et al., 2022). 0.2% chlorhexidine mouthwash used three times daily can effectively treat RAS. Minocycline, typically administered as a mouthwash, can effectively alleviate the pain caused by RAS. A novel herbal mouthwash was developed using *Artemisia argyi*, *Chrysanthemum morifolium*, *Lonicera japonica*, *Angelica dahurica*, and *Polygonum aviculare* as ingredients. The evaluation revealed that the healing time and ulcer area accelerated the healing process of the oral ulcer mucosa in rats (Wu et al., 2023). Owing to salivary washout, these preparations used to treat oral mucosal diseases such as RAS and Behçet’s disease in liquid form remain in the mouth for a very short period, and prolonged use can result in taste disturbances, temporary staining of the teeth and tongue, and disruption of the normal flora in the oral cavity (Senusi et al., 2020; Ni et al., 2022; Wu et al., 2023).

### 4.2 Powders

Powdered formulations refer to solid dosage forms ground into a powder that can be absorbed by the oral mucosa and prepared according to traditional Chinese medicine combinations. Currently, the powders used for treating RAS include watermelon frost and Bingpeng powder, with watermelon frost being the most extensively researched and applied. Its active components, such as luteolin, rhein, and emodin, have been proven to demonstrate definite therapeutic efficacy against RAS (Wu et al., 2024). The therapeutic efficacy of powders depends on both their adhesion strength and quantity at ulcer sites. Owing to the constant flow of oral saliva, ulcers are easily washed away, leading to a reduced drug concentration and compromised treatment outcomes (Suharyani et al., 2021).

### 4.3 Ointment

Ointment is a semisolid dosage form that is applied directly to the surface of the ulcer. It relieves pain and reduces inflammation owing to drug penetration through a physical barrier. Triamcinolone acetonide oral ointment is a prescription steroid medication used to treat dermatitis, mucositis, and mouth ulcers (Ono et al., 2024). Poor retention at the ulcer site and a short drug retention time require frequent application (Suharyani et al., 2021).

### 4.4 Gel

Gels are three-dimensional network structures formed through interactions between colloidal particles and aqueous, non-aqueous, hydroalcoholic, or alcohol solutions. They possess pain-relieving, healing-promoting, and wound-protective properties (Suharyani et al., 2021). They are typically applied directly to ulcer surfaces, where they exert local therapeutic effects through contact with mucous membranes. They can be loaded with antimicrobial agents, anti-inflammatory drugs, analgesics, or bioactive substances according to specific requirements. Raman et al. evaluated the efficacy of curcumin gel in RAS patients and reported that it positively influenced ulcer size, number, pain levels, and healing (Raman et al., 2020).

### 4.5 Adhesive agent

Currently, commercial mouthwashes, powders, ointments, and recombinant human epidermal growth factor gels are used to accelerate the healing of oral ulcers. However, their therapeutic efficacy is low because of their short retention time (<2 h) on the mucosal surface in the highly moist and dynamic environment of the oral cavity. To address this limitation, locally adherent delivery systems have been developed. These offer several advantages, including intimate contact between the drug formulation and oral mucosa, extended retention time, and controlled-rate drug release to specific target sites.

Oral mucosal adhesive patches are solid dosage formulations that contain drugs that are absorbed directly into the oral mucosa. These materials include polymer membranes and 3D-printed structures, which may be composed of monolayers, multilayers, and sandwich forms (Paderni et al., 2012; Chen H. et al., 2023). Adhesive patches provide physical protection for ulcers to alleviate pain, attach securely to the mucosa to provide sustained drug release, bypass the first-pass effect, and enhance bioavailability. A simple preparation method was utilized to explore a self-stabilized and water-responsive binary synergistic patch made of the coenzyme polymer poly (α-lipoic acid) (PolyLA) and the polymer poly (α-lipoic acid sodium) (PolyLA-Na) for enhancing mucosal adhesion and accelerating the healing of oral ulcers in a humid oral environment. When the patch is applied to mucosal ulcers, the water triggers the sustained release of antioxidant bioactive small molecules in the patch, which play antibacterial, anti-inflammatory, and antioxidant roles to regulate the microenvironment of the wound, thereby greatly improving the efficacy of the treatment of oral ulcers (Cui et al., 2023). An *in vitro* release study of triamcinolone acetonide was conducted using a novel double-layer adhesive film. Experimental films of hydroxypropylmethylcellulose (HPMC), polyvinyl alcohol (PVA), and polyvinylpyrrolidone (PVP) were prepared using the solvent casting method, and ethylcellulose (EC) was used as a backing layer to load triamcinolone acetonide (TA). These formulations improved patient compliance and achieved a sustained release of TA, which had a positive effect on ulcer healing (Alhallak et al., 2023). A study reported the successful development of a bilayer oral film called the C-P-G film, which consists of an adhesive layer of Sodium Carboxymethyl Cellulose (CMC), PVP, glycerol, and a hydrophobic layer of zein. This film has an extremely thin and light appearance, achieves strong and long-lasting adhesion to moist oral mucosa and optimal biocompatibility, thereby achieving effective treatment of oral ulcers (Yan et al., 2024). Some of the shortcomings of traditional preparation techniques for this film are expected to be addressed with the advent of 3D printing technology, which can be employed to manufacture a range of customized oral veneers to meet different patient needs, given the robust maneuverability and precise printing of complex structures enabled by this technology (Eleftheriadis et al., 2019).

Among numerous biomaterials, hydrogels have garnered significant attention in the field of RAS healing because of their exceptional hydrophilicity, biocompatibility, and degradability. Hydrogels are three-dimensional network materials formed by cross-linking natural or synthetic polymers (Moussa et al., 2023; Cheng et al., 2025). Research indicates that the preparation of hydrogels using licorice and hydroxyethyl cellulose (HEC) as green gelling agents enables the slow release of active compounds from licorice. This creates a controlled-release system that inhibits the growth and reproduction of microorganisms such as bacteria and fungi. Hydrogels containing 30% licorice extract and 4% hydroxyethyl cellulose (HEC) significantly promote oral traumatic ulcer healing (Moussa et al., 2023). Unlike skin wounds, the oral cavity maintains a persistently moist environment due to the presence of abundant saliva. Therefore, when hydrogels are designed for RAS treatment, incorporating or enhancing multiple functionalities—such as moist adhesion capabilities—to adapt to this unique physiological setting is essential (Zhang W. et al., 2025). Wang et al. developed an adhesive hydrogel patch (AHP) composed of quaternized chitosan, aldehyde-functionalized hyaluronic acid, and a tridentate complex of protocatechuic aldehyde with Fe^3+^ (PF). AHP has tunable mechanical properties, self-healing capabilities, and moist adhesion to the oral mucosa. By controlling the formulation of AHP, PF is released from AHP in a time-dependent manner, effectively promoting ulcer healing (Wang Y. et al., 2024). A Janus-like patch named ANSB was developed, inspired by the multilayered and asymmetric structure of natural mucosa, featuring a durable adhesive layer and a lubricating layer. By overcoming the saliva barrier and leveraging covalent cross-linking between tissue surface amine groups and N-hydroxysuccinimide esters, the adhesive layer composed of gelatin and acrylic achieves rapid (≤30 s), strong (≥45 kPa), and durable (≥8 h) adhesion to moist oral tissues (Zhang et al., 2024a). Sprayable hydrogels for sealing moist, dynamic, and concealed wounds within the body (e.g., polyethylene glycol (PEG)-based hydrogels) achieve extensive coverage of ulcerated surfaces through their unique spray-on delivery method. Low swelling properties and fatigue resistance ensure sustained wound coverage. Sprayable polyethylene glycol (PEG)-based hydrogels not only exhibit outstanding fatigue resistance and low swelling characteristics but also demonstrate excellent biocompatibility and antimicrobial activity, offering a novel technical approach for treating oral ulcers (Zhang et al., 2024b).

Currently, most research on oral adhesive patches remains in the laboratory stage, with only a few materials successfully achieving clinical translation. The oral environment—moist, open, and in constant motion—poses significant challenges for existing adhesive materials. However, excessive adhesion or prolonged retention time may lead to incomplete material degradation, potentially interfering with patient eating and speech and ultimately reducing medication compliance. Therefore, establishing an optimal performance standard that balances effective adhesion with a positive user experience is imperative. Future research should focus on advancing the translation of fundamental research findings into clinical applications to drive substantive progress in this field (Zhao, 2023; Zhang W. et al., 2025).

### 4.6 Microneedle

A microneedle (MN) is an innovative form of solid drug delivery that significantly improves drug delivery efficiency through the use of micron-sized (<1,000 μm in length) needles that can be precisely prepared from a variety of biocompatible materials with diverse geometric configurations (Zhuo et al., 2025). In recent years, MN technology has become a research hotspot in the field of drug delivery because of its outstanding advantages of minimal invasiveness, high efficiency, and painlessness. The core mechanism of action of microneedling is to create controlled microchannels in the stratum corneum of the skin, which significantly increases the transdermal absorption rate of drugs, facilitating their efficient entry into the body circulation. Since MN penetrate only the stratum corneum without touching deeper nerve endings, the process of drug delivery is almost painless (Sartawi et al., 2022; Andranilla et al., 2023; Qiang et al., 2023; Babu et al., 2024; Huang et al., 2024).

In the field of oral ulcer (RAS) treatment, microneedle technology has unique advantages. Compared with traditional topical drug delivery, MN can effectively address key issues such as the short duration of drug action, insufficient effective concentration, and single efficacy. Current oral ulcer research focuses primarily on developing soluble microneedles. These needles can simultaneously carry one or more synthetic or natural drugs to achieve therapeutic effects, which can completely dissolve in the skin, ensuring efficient drug delivery and avoiding the problem of the disposal of sharp medical waste after use.

Rapid-release soluble MN achieves rapid drug release through rapid dissolution, resulting in rapid pain relief and the promotion of ulcer healing. These microneedles are usually prepared using the solvent casting method and are manufactured using solvent-filled (usually water) micromolds with matrix materials, such as hyaluronic acid (HA), carboxymethyl chitosan (CMC), polyvinylpyrrolidone (PVP), polyvinyl alcohol (PVA), poly (lactic acid)-glycolic acid copolymer (PLGA), and sericin protein. Soluble hyaluronic acid (HA) microneedle patches (BSP-BDP@HAMNs), loaded with betamethasone sodium phosphate (BSP) and betamethasone dipropionate (BDP), effectively suppress inflammation and promote wound healing, and compared to traditional delivery methods, they achieve painless penetration of ulcer surfaces with sustained action, thereby enhancing both comfort and efficacy in oral ulcer treatment (Guo et al., 2023). To treat oral ulcers, a soluble HA/Bletilla polysaccharide (BSP)-based microneedle loaded with triamcinolone acetonide (TA) was developed to enhance TA bioavailability, which achieves efficient penetration and rapid dissolution within 3 min, demonstrating excellent biocompatibility and anti-inflammatory properties, and resulted in a significant reduction in both ulcer area and levels of inflammatory mediators such as TNF-α and CD31 following treatment (Qu et al., 2023). Silk fibroin (SF) MN loaded with Lipopolysaccharide (LPS)-preconditioned bone marrow mesenchymal stem cells and their secreted exosomes (LPS-Pre-Exos) and Zeolitic imidazolate framework-8 (ZIF-8) possess good anti-inflammatory and antimicrobial properties, promote oral ulcer healing, and exhibit good histocompatibility (Ge et al., 2024). An innovative multifunctional magnesium metal organic skeleton embedded with a hyaluronic acid-soluble microneedle patch with curcumin that integrates slow drug release, multiple therapeutic methods, and natural biomaterials for the treatment of recurrent oral ulcers was developed, and for the first time, a bioactive magnesium-based metal organic skeleton loaded with a natural biomaterial, curcumin, was used for the treatment of oral mucous membrane diseases. This process results in the slow release of drugs, causing the gradual decomposition of nanoparticles, which significantly improves therapeutic efficiency (Liu J. et al., 2024).

**FIGURE 5:**
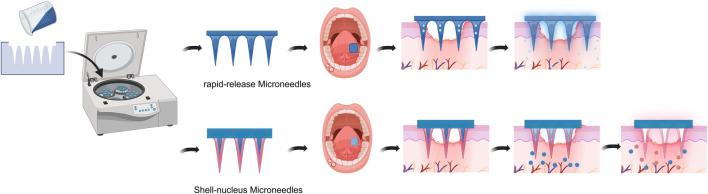
Schematic representation of the rapid release of soluble MNs and shell-nucleus MNs in the RAS. rapid-release soluble MNs rapidly dissolve their tip matrix upon contact with RAS, promptly releasing the drug loaded onto the tip. Shell-nucleus MNs rapidly dissolve their core matrix upon contact with RAS, releasing the drug within the core, while the shell matrix slowly swells to achieve sustained release of the drug within the shell. Created in BioRender. Xiangran, k. (2026) https://BioRender.com/siwy0jv.

To address the special challenges of transmucosal drug delivery, a slow-release soluble MN with a shell-nucleus structure has been developed. This novel design provides a more optimized solution for RAS treatment, in which different drugs are loaded at different layers of the microneedle (shell and nucleus) to achieve the controlled release of drugs in a time-dependent manner. Compared with traditional single microneedle patches, shell-nucleus microneedles can effectively penetrate the oral mucosa, release multiple drugs in an orderly manner, and completely dissolve within the tissue to achieve a synergistic therapeutic effect. A well-designed bifunctional core-shell MN patch was prepared to treat oral ulcers. The outer shell at the tip of the microneedle is made of hyaluronic acid (HA) and loaded with lidocaine, whereas the inner core, consisting of gelatin methyl methacrylate (GelMA), encapsulates dexamethasone. The HA shell dissolves rapidly upon application, facilitating the immediate release of lidocaine, which induces anesthesia at the ulcer site, thereby reducing the associated pain sensation. Moreover, GelMA adds mechanical strength to ensure the sustained release of dexamethasone. In this way, the rapid healing of mouth ulcers can be accelerated (Tang et al., 2024). The novel hyaluronic acid methacryloyl (HAMA)-HA-PVP MN rapidly penetrated the mucosa, effectively dissolving and releasing the drug for sequential drug delivery. The drug loaded in the HA fraction is released rapidly as the HA layer dissolves. The drug loaded in the crosslinked HAMA is subsequently released slowly as the HAMA swells into the tissue fluid. Compared with monolayer MNs, HAMA-HA-PVP MNs can be used as a bilayer drug reservoir for controlled drug release, effectively releasing the drug from the MN. *In vitro* and *in vivo* biosafety evaluation tests revealed that HAMA-HA-PVP MNs are minimally invasive, reversible, nontoxic, and safe for use, and the drug-loaded HAMA-HA-PVP MNs performed excellently in the treatment of oral ulcers in rats as test subjects (Meng et al., 2023). This drug delivery system can, therefore, serve as an efficient, multi-permeable, mucosal, and needle-free alternative for biomedical applications (Figure 5).

Although animal studies indicate that microneedle technology has promising safety and efficacy potential in treating oral ulcers, its clinical translational feasibility and actual efficacy in humans remain to be further validated, as current evidence is still limited to the animal experimental stage (Liu et al., 2021; Kong et al., 2024).

### 4.7 Nanomaterials

The emergence of novel nanomaterials has provided new ideas for drug delivery (He et al., 2021). Nanodrug delivery technology effectively overcomes the many limitations of traditional drug delivery methods, as it increases the water solubility of drugs, enhances drug bioavailability, improves targeting, and reduces toxic side effects. In the field of oral ulcer treatment, nanoformulations can provide significantly enhanced therapeutic effects and help patients recover faster by virtue of their excellent drug-carrying capacity, *in vivo* stability, and controlled release characteristics. Currently, a variety of nanodrug delivery technologies are used for oral mucosal drug delivery and have shown a growing trend annually; however, liposomes, nanoemulsions, polymer nanoparticles, nanofibers, etc., can be effectively used in the field of wound healing (Zupancic et al., 2015; Bhadauria and Malviya, 2022; Cocoş et al., 2024; Liu S. et al., 2024).

#### 4.7.1 Liposomes

As one of the most established nanodelivery systems, liposomes consist of bilayer vesicle structures composed of natural or synthetic phospholipids, with excellent biocompatibility and tissue tolerance (Zupancic et al., 2015; Rahma et al., 2025). In an *in vitro* study investigating the efficacy of wheat germ agglutinin (WGA)-conjugated liposomes loaded with amoxicillin for the treatment of oral ulcerative lesions, compared with untreated controls and free amoxicillin, the WGA-conjugated liposomes significantly reduced oral cell damage by promoting rapid binding to specific proteins associated with oral cells (Wijetunge et al., 2020). EL-Wakeel and Dawoud conducted a randomized controlled clinical trial to evaluate the efficacy of topical insulin liposome gel in treating oral ulcers. Eighty participants with mild oral ulcers received either topical insulin liposome gel or placebo gel (once daily) for 6 days. Participants treated with insulin-liposome gel underwent outcome measurements via the visual analog scale (VAS) on days 1, 2, 3, 4, and 6, along with the Oral Health Impact Profile-14 (OHIP-14) assessment on day 6. The mean duration of symptoms was significantly shorter in the treatment group than in the placebo group (P = 0.001). After 6 days, the OHIP-14 scores were significantly lower in the treatment group than in the placebo group (P = 0.001). The reduction in pain scores demonstrated the efficacy of insulin-liposome formulations in treating typical discomfort associated with oral ulcers (El-Wakeel and Dawoud, 2019).

#### 4.7.2 Nanoemulsions

A nanoemulsion is a transparent or semitransparent dispersion system composed of droplets with particle sizes less than 200 nm and offers the advantages of easy preparation and rapid drug release. Its small particle size effectively prevents problems such as sedimentation and flocculation, which are common in traditional emulsions (Singh et al., 2017; Alissa et al., 2024). A study on the therapeutic effect of a psyllium standard extract nanoemulsion on oral ulcers in Wistar rats revealed that the 5% Plantago major standardized extract (PMSE) nanoemulsion achieved complete epithelialization in 66.7% of the oral lesions and reduced inflammation in 88.3% of the lesions. The 5% PMSE nanoemulsion demonstrated excellent penetration into the wound site, promoting ulcer healing (Jahanimoghadam et al., 2024).

#### 4.7.3 Polymer nanoparticles

These solid colloidal particles have a particle size of 10–1,000 nm and can be prepared from natural or synthetic polymers. Polyethylene glycolized poly (hexadecyl cyanoacrylate) nanoparticles effectively facilitate drug transmembrane transport and reduce toxic side effects while maintaining the therapeutic dosage (Zupancic et al., 2015; Rezazadeh et al., 2021; He et al., 2021). A study on the efficacy of the newly developed doxycycline hydrochloride-loaded polyglutamic acid/tannic acid nanoparticle system for treating rat oral ulcers demonstrated that the retention rate of the drug-loaded nanoparticles in ulcer tissue was 17 times greater than that of the free drug. Exhibiting antibacterial and immunomodulatory effects, this system significantly accelerated the healing of oral ulcer wounds. These doxycycline-loaded polyglutamic acid/polyglutamic acid/tannic acid nanoparticles represent a novel and effective therapeutic strategy for oral ulcers (He et al., 2023).

#### 4.7.4 Nanofibers

Nanofibrous membranes are novel drug delivery vehicles for programmed drug release and long-lasting local drug delivery (Torres-Martínez et al., 2019; Zhou et al., 2022). Zhang developed fast-dissolving drug delivery systems using honey and acetylsalicylic acid-embedded poly (vinyl alcohol) (PVA) nanofibers based on natural deep eutectic solvents (DESs). An *in vivo* study revealed that PVA–DES–honey nanofibers accelerated the wound healing process and improved the wound healing rate on rat skin to 85.2% after 6 days of surgery (Zhang et al., 2021). Chang et al. prepared an adhesion membrane composed of calcium ion-crosslinked carboxymethyl cellulose nanofibers and alginate loaded with two drugs—dexamethasone (DXM) and dyclonine hydrochloride (DYC)—for treating rat oral ulcers. The fiber membrane exhibited strong tissue adhesion, resisted deformation caused by frequent oral movements, demonstrated excellent water resistance, and possessed good biocompatibility. *In vivo* experiments in rats demonstrated its effective therapeutic effect on oral ulcers (Chang et al., 2024).

Ensuring favorable local biocompatibility and safety within the oral mucosa is a core prerequisite for the clinical translation of novel drug delivery systems. Safety assessments typically revolve around a series of typical endpoint indicators, primarily including: 1) Local irritation (e.g., presence of erythema, edema, or erosion in the mucosa, quantified via irritation indices); 2) Sensitization (evaluated through methods such as maximization tests); 3) Cytotoxicity (evaluated via *in vitro* cell culture); and 4) Systemic toxicity (assessed through histopathological analysis of major organs). For novel adhesives, such as the Polyla-Na/Polyla patch developed by Cui et al. and the C-P-G adhesive film reported by Yan et al., comprehensive evaluations demonstrated no significant cytotoxicity, mucosal irritation, or systemic toxicity. Furthermore, these adhesives did not induce allergic reactions in animal models (Cui et al., 2023; Yan et al., 2024). For microneedle (MN) technology, safety endpoints particularly emphasize pain-free administration (assessed via visual analog scales) and short-term reactions to minimally invasive punctures. For instance, a study involving 30 volunteers confirmed that micro-needle implantation at multiple oral sites elicited significantly lower pain than injection needles and showed no statistically significant difference compared to a negative control (Santos et al., 2021). Guo et al. also observed only mild, rapidly resolving hyperemia at micro-needle puncture sites in a rat model, with no significant histological inflammation. These findings consistently indicate that the novel formulations demonstrate good safety and biocompatibility within existing evaluation systems. However, it must be noted that most current evidence remains derived from preclinical studies. Future efforts should prioritize advancing clinical translational research aimed at validating human tolerability and compliance.

Most preclinical evidence for adhesion-based formulations, microneedles, and nanodelivery technologies originates from rat ulcer models. While these models effectively simulate common ulcer healing processes and are widely used for drug efficacy screening, they cannot fully represent the complex processes of human idiopathic RAS. Rigorous randomized controlled trials in patients with idiopathic RAS are required to validate their therapeutic efficacy.

Currently, research on oral mucosal delivery systems based on adhesive patches, microneedles, and nanodelivery technologies remains in the fundamental research stage, lacking systematic comprehensive quality evaluation. This has to some extent constrained the development and clinical translation of novel oral mucosal delivery formulations utilizing nanodelivery technology-based delivery systems (Chen S. et al., 2023). Table 6 summarizes novel delivery systems, preparation methods, matrix, loaded drugs, adhesion strength and duration, biocompatibility, efficacy.

**TABLE 6:** Novel delivery systems, preparation methods, matrix, loaded drugs, adhesion strength and duration, biocompatibility, efficacy.

Drug delivery system	Preparation	Matrix	Drug	Adhesion		Biocompatible	Therapeutic efficacy			Ref
				Strength	Time		Comparator	Model	Promote ulcer healing	
Adhesive patch	Solvent Evaporation	α-Lipoic acid (LA), NaOH aqueous solution	Unloaded Drug	60 kPa	24 h	In vitro and *in vivo* (rat)	Control, com-mercial chitosan film-treated	rat	Positive	Cui et al. (2023)
Adhesive film	Solvent casting method	Hydroxypropyl methylcellulose (HPMC), polyvinyl alcohol (PVA), polyvinyl pyrrolidone (PVP), ethyl cellulose (EC)	Triamcinolone acetonide (TA)	4.03 ± 0.90N	24 h	Vitro	No studies available			Alhallak et al. (2023)
	Solvent Evaporation	Sodium carboxymethylcellulose (CMC), PVP, glycerol Zein	Dexamethasone (Dex)	60 kPa. Porcine buccal mucosa 5 kPa	152min	In vitro and *in vivo*	Control, the commercially available drug (oral Patch 3 group)	rat	Positive	Yan et al. (2024)
Adhesive patch	3D printing	Chitosan (CS),PVA, EC, Xylitol (XYL)	Dex	Superior adhesion compared to commercial formulations		In vivo (rat)	Control	rat	Positive	Chen et al. (2023a)
Adhesive hydrogel	​	Quaternary ammonium salt of chitosan, aldehyde-functionalized hyaluronic acid, and a tridentate complex of protocatechualdehyde and Fe3+ (PF)	Unloaded Drug	Pigskin and oral mucosa tissue of the rats3kpa	No studies available	In vitro	Control	rat	Positive	Wang et al. (2024b)
		Gelatin, acrylic acid	Unloaded Drug	≥45 kPa	≥8 h	In vitro and *in vivo* (rat)	Control, propolis oral film, Jasland oral Patch	rat	Positive	Zhang et al. (2024a)
Dissolvable microneedle	One-step casting method 、Two-step casting method、three-step casting method	Hyaluronic acid (HA)	Dexamethasone acetate, vitamin C and tetracaine hydrochloride	No studies available		In vitro	Control, HA MN, watermelon frost	rat	Positive	Wang et al. (2023)
		HA, hydroxypropyl trimethyl ammonium chloride chitosan (HACC)	Dexamethasone and basic fibroblast growth factor	No studies available		In vitro	Control, HA MN, HA/HACC MN, HA/HACC@DXMS MN, HA/HACC@bFGF MN	rat	Positive	Zeng et al. (2023)
		HA	Betamethasone 21-phosphate sodium (BSP) and betamethasone 17,21-dipropionate (BDP)	No studies available		In vitro and *in vivo* (rat)	Control, triamcinolone acetonide dental paste (Ning Zhi Zhu®), HAMN, BSP-BDP@HA film	rat	Positive	Guo et al. (2023)
		HA	TA, epidermal growth factor (EGF), Zeolitic imidazolate framework-8 (ZIF-8)	No studies available		In vitro and *in vivo* (rat)	Control, HA MN, T-HA MN TE-HA MN	rat	Positive	Ge et al. (2023)
		Gelatin methacryloyl (GelMA), HA	Basic fibroblast growth factor (bFGF), dexamethasone, ZIF-8	No studies available		In vitro and *in vivo* (rat)	Control, HA MN, GelMA/HA MN, GelMA@bFGF/HA MN, GelMA@bFGF/HA@DXMS MN	rat	Positive	Yu et al. (2024)
		Silk fibroin (SF)	Lipopolysaccharide (LPS)-preconditioned bone marrow mesenchymal stem cells and their secreted exosomes (LPS-pre-Exos), zeolitic imidazolate framework-8 (ZIF-8)	Approximately 1.5 times that of commercial oral patches	No studies available	In vitro and *in vivo* (rat)	Control, SF MN Single-loaded LPS-pre-Exos MN	rat	Positive	Ge et al. (2024)
		HA	curcumin (CUR)-loaded Porous magnesium metal–organic framework (Mg-MOF)(MC), ε -poly-l-lysine (EPL)	No studies available		In vitro and *in vivo* (rat)	Control, HA MN, EPL MN, MC MN	rat	Positive	Liu et al. (2024a)
		HA, Bletilla striata polysaccharide (BSP)	TA	No studies available		In vitro and *in vivo* (rat)	Control, triamcinolone acetonide dental paste (Ning Zhi Zhu®), HA/BSP MNs	rat	Positive	Qu et al. (2023)
		Hyaluronic acid methacryloyl (HAMA), HA, PVP	Betamethasone (BT), lidocaine	No studies available		In vitro and *in vivo* (rat)	Control Single-loaded BTMN Single-loaded lidocaine MN	rat	Positive	Meng et al. (2023)
		HA,GelMA	Lidocaine, dexamethasone	No studies available		In vitro	Control MN, dexamethasone	rat	Positive	Tang et al. (2024)
		HA	recombinant bovine basic fibroblast growth factor (rbFGF), cetylpyridinium chloride (CPC)	No studies available		In vitro	Control HA MN, CPC HA MN, rbFGF HA MN CPC gargle combined with rbFGF gel	rat	Positive	Yin (2021)
Polymer nanoparticles	Esterification reaction	Polyglutamic acid, tannic acid	Doxycycline hydrochloride (DCH)	No studies available		In vitro	Control, DCH solution	rat	Positive	He et al. (2023)
Nanofibers	Casting method	CMC,alginate	Dexamethasone, dyclonine hydrochloride (DYC)	Average adhesion force of 5.5 ± 1.4 KPa	No studies available	In vitro	Control	rat	Positive	Chang et al. (2024)

## 5 Challenges

### 5.1 Limitations of existing clinical treatment approaches

Current clinical management strategies for RAS exhibit significant shortcomings. While first-line topical corticosteroids effectively reduce inflammation, long-term use may cause adverse reactions such as mucosal atrophy and offer limited efficacy in preventing recurrence. Anesthetics provide only temporary pain relief without significantly promoting ulcer healing, whereas prolonged antibiotic use may disrupt the oral microbiome balance and even induce secondary infections. Systemic corticosteroids and immunomodulators are effective at reducing recurrence, but their potential systemic side effects limit their long-term use in special populations, such as children and pregnant women. Current clinical treatments employ formulations such as mouthwashes, powders, and ointment gels. However, owing to saliva flushing and oral motility, these medications struggle to remain in place long term. This situation underscores the urgent need for novel therapies that combine sustained efficacy, safety, and curative potential.

### 5.2 Core challenges in natural medicinal product development

Owing to their favorable safety profiles, reduced side effects, and high potential efficacy, natural Chinese herbal medicines and their active components offer new possibilities for RAS treatment. However, the path from “potential” to “product” faces multiple bottlenecks. First, the specific molecular targets and pathways by which most complex natural herbal components treat RAS remain incompletely elucidated. Second, many highly active compounds (e.g., curcumin and quercetin) suffer from poor water solubility, chemical instability, and low oral bioavailability, severely limiting their ultimate therapeutic efficacy. Third, existing studies are predominantly small-sample or open-label trials and lack support from large-scale, double-blind randomized controlled trials (RCTs). This results in low-grade evidence, making adoption in clinical guidelines challenging. Finally, while no reports of severe side effects from natural Chinese herbal medicines or their active components exist, considering potential drug interactions when initiating treatment alongside other medications is crucial.

### 5.3 Core challenges in delivery system innovation

**FIGURE 6:**
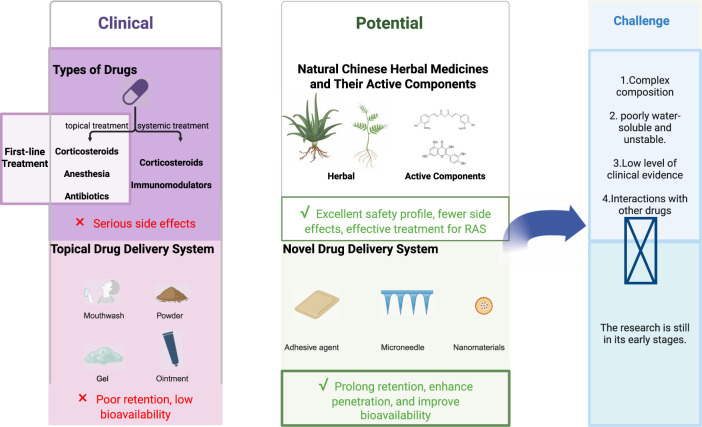
Current status of RAS research in drug therapy: Limitations of current clinical treatments, key challenges in advancing natural medicines and novel delivery systems from laboratory to clinical practice. Created in BioRender. Xiangran, k. (2026) https://BioRender.com/wwnkj9h.

Despite demonstrating significant potential in prolonging retention, enhancing penetration, and improving bioavailability in laboratory settings, novel drug delivery systems such as mucosal-adherent hydrogels, nanomedicines, and microneedles remain in the early stages of clinical translation. The “translation gap” manifests primarily in the following aspects: insufficient research on the long-term safety and therapeutic efficacy of these complex systems; challenges in cost and control for large-scale production; and the regulatory review pathways and quality standards systems for such innovative dosage forms that have yet to be fully established. Figure 6 summarizes the current status of RAS research in drug therapy: limitations of current clinical treatments, key challenges in advancing natural medicines and novel delivery systems from laboratory to clinical practice.

## 6 Future outlook

Future research should focus on deeply integrating the therapeutic advantages of natural medicines with the precision and sustained-release characteristics of advanced delivery systems. We anticipate that next-generation RAS treatment strategies will no longer be a mere accumulation of individual drugs or technologies but rather an integrated solution. By designing smart responsive delivery systems (such as microenvironment-responsive hydrogels and bioadhesive nanoparticles), we can increase the solubility of natural active ingredients, deliver them precisely to ulcer sites, and achieve on-demand, sustained release at the lesion site. This approach promises a qualitative improvement in pain control and accelerated healing.

Ultimately, the success of this strategy hinges not only on technological breakthroughs but also on rigorous clinical validation and personalized treatment concepts. By conducting large-scale, rigorously designed clinical trials to generate high-level evidence demonstrating superior efficacy and building upon this foundation to develop personalized treatment plans tailored to each patient’s ulcer type, recurrence frequency, and systemic condition, we aim to increase RAS treatment from temporary symptom management to a new era of long-term relief and even a cure. This will fundamentally improve the long-term treatment experience and quality of life for the vast majority of RAS patients.
